# Low dose of interferon-α improves the clinical outcomes of docetaxel in patients with castration-resistant prostate cancer: A pilot study

**DOI:** 10.3892/ol.2013.1653

**Published:** 2013-11-01

**Authors:** YUN-FEI LI, QIN-ZHANG WANG, TAO-TAO ZHANG, LEI LI, JIANG-PING WANG, GUO-FU DING, DA-LIN HE

**Affiliations:** 1 Department of Urology, First Hospital of Xi’an Jiaotong University, Xi’an, Shaanxi 710061, P.R. China; 2 Department of Urology, First Hospital of Shihezi University, Shihezi, Xinjiang 832008, P.R. China; 3 Department of Urology, Renmin Hospital, Hubei University of Medicine, Shiyan, Hubei 442000, P.R. China

**Keywords:** castration-resistant prostate cancer, docetaxel, interferon-α-2b

## Abstract

The aim of this study was to test whether a low dose of interferon-α-2b (IFN-α2b) enhances the clinical outcome of docetaxel (DXT) in patients with castration-resistant prostate cancer (CRPC). A prospective controlled trial of 40 CRPC patients receiving 5 mg of prednisone twice daily was conducted, where patients were randomly assigned to be administered 75 mg/m^2^ DXT plus 3 mIU/m^2^ IFN-α2b (group A, n=20) or 75 mg/m^2^ DXT alone (group B, n=20). The prostate-specific antigen (PSA) response, tumor response, progression-free survival (PFS) and overall survival (OS) were evaluated. There was no statistically significant difference in PSA response rate between groups A and B (65 vs. 47.4%, P=0.341). The tumor response rate in group A was significantly greater compared with that in group B (55 vs. 21.1%, P=0.048). The median PFS was longer in group A compared with that in group B (10 vs. 8 months, P=0.043). There was no statistically significant difference in median OS between the two groups (19 vs. 17 months, P=0.348), but one patient displayed a complete tumor response in group A. In groups A and B, transient grade 3 to 4 neutropenia was observed in nine and six patients, grade 3 to 4 anemia was observed in three and five patients, and grade 3 to 4 general fatigue was observed in four and one patient(s), respectively. The proportion of patients with grade 3 to 4 toxicity was not statistically different between the two groups. A low dosage of IFN-α2b may improve the antitumor activity of DXT with an acceptable toxicity profile in patients with CRPC.

## Introduction

Early-stage prostate cancer can be cured by radical surgery or radiation therapy. However, ~10–20% of men with prostate cancer have metastatic disease, and many others develop metastases despite primary treatment (1,2). Although the majority of patients with advanced metastatic disease initially respond to conventional androgen deprivation with medical or surgical castration, the response to hormonal treatment lasts for a median duration of 18–24 months. Thus, most patients eventually become resistant to therapy and develop hormone refractory prostate cancer, also known as castration-resistant prostate cancer (CRPC) (2).

Treatment of CRPC is currently a challenge. Options for improved survival in patients with CRPC are limited, but docetaxel (DXT) chemotherapy is the clearly established treatment (3). While DTX chemotherapy has shown success with overall survival improved by 2.5 months compared with that of mitoxantrone-based therapy, only ~48% of patients respond, and drug resistance rapidly develops to treatment with a combination of DXT and prednisone (4,5). Two important mechanisms of tumor resistance, which may be exploited therapeutically, are the overexpression of Bcl-2 and the loss of p53 function, both of which contribute to the resistance of CRPC to DXT (5–10).

It has been demonstrated that interferon-α-2b (IFN-α2b) decreases the expression of Bcl-2 and increases the expression of p53 (11,12). Similarly, IFN-α2b has been shown to promote the effects of DTX chemotherapy *in vitro*(13). A low dosage of IFN-α2b showed antitumor activity in prostate cancer patients in a recent study with a follow-up period of >10 years (14). In addition, IFN-α2b alone or in combination with chemotherapy drugs has been proven safe and effective in various metastatic malignant tumors (15,16). However, though a high dosage of IFN-α2b alone demonstrates antitumor activity, its toxicity is intolerable for use in CRPC (17). Conversely, toxicity levels with a moderate dosage of IFN-α2b alone are acceptable, whereas the efficacy is limited (18).

These facts led us to hypothesize that IFN-α2b may be able to expand the effects of DXT chemotherapy. In this pilot study, we conducted a prospective analysis to evaluate the efficacy and toxicity of this regimen in patients with CRPC.

## Patients and methods

### Patients

From January 2007 to September 2009, 40 patients from the Department of Urology, First Hospital of Shihezi University (Shihezi, China) with CRPC were enrolled in this study. To be eligible for this study, patients had to have an Eastern Cooperative Oncology Group (ECOG) performance status of 0 or 1 (http://ecog.dfci.harvard.edu/general/perf_stat.html), and evidence of progressive metastatic disease despite androgen deprivation therapy. Patients were required to have a serum testosterone concentration of <50 ng/dl and prior treatment with maximum androgen blockade with evidence of treatment failure. Patients were required to have a measurable soft tissue lesion. Patients had to have adequate function of bone marrow, liver, heart, kidney and lung, defined as white blood cell count ≥4,000/mm^3^, granulocytes ≥2,000/mm^3^, platelet count ≥100,000/mm^3^, bilirubin ≤1.5 mg/dl, alanine aminotransferase and aspartate aminotransferase ≤2-fold the institutional upper limit of normal, creatinine ≤2.0 mg/dl or a calculated creatinine clearance ≥50 ml/min, left ventricular ejection fraction ≥50% as demonstrated by echocardiography, and forced expiratory volume in one second/forced vital capacity ≥70%. Patients were required to have no history of myocardial infarction within 6 months of study entry, and no history of deep venous thrombosis.

The study was performed after approval by the Human Investigations Committee of the Medical College of Shihezi University (Shihezi, China). Informed consent was obtained from each patient.

### Medical protocol

Throughout the treatment period, patients in group A received DXT (75 mg/m^2^) by intravenous administration over 60 min on day 1, IFN-α2b (3 mIU/m^2^) was subcutaneously injected on days 16, 18 and 20; and oral prednisolone (5 mg) was administered twice daily on days 1 to 21, on each 21 day cycle. Patients in group B were treated as abovementioned, but were not administered IFN-α2b. For the first cycle of treatment, all patients were hospitalized for 8–10 days to check renal and liver functions and to observe adverse events. For the second course of treatment, all patients were treated on an outpatient basis. The treatment was continued until the disease progressed or unacceptable adverse events occurred. Patients were hospitalized if grade 3 to 4 neutropenia was observed, and granulocyte-colony stimulating factor (G-CSF) was used. The dose of DXT was reduced by 10 mg/m^2^ in subsequent treatment cycles if granulocytes remained at ≤2,000/mm^3^ after the G-CSF treatment in patients with grade 3 to 4 neutropenia.

### Treatment evaluation

The endpoints of this study comprised prostate-specific antigen (PSA) response, tumor response, the time to PSA progression, and toxicity. PSA response was defined as a reduction of at least 50% in the baseline levels for 4 weeks. PSA progression was defined as a rise in PSA levels exceeding 25% of the baseline level. The tumor response was documented using radiographs according to response evaluation criteria in solid tumors (RECIST) (19) after four cycles of therapy (performed by a single radiologist). Tumor complete response was defined as the disappearance of all evidence of a tumor and all symptoms of cancer for at least 12 weeks. Partial response was defined as ≥50% decrease in the sum of the products of diameters of all measured lesions persisting for at least 12 weeks. Tumor progression was defined as a rise in the diameter exceeding 25% of the baseline level. Progression-free survival (PFS) was defined from the date of the first chemotherapy treatment to the first occurrence of PSA and tumor progression. Overall survival (OS) was defined from the date of the first chemotherapy treatment to the date of death from any cause. The Cancer Institute Common Toxicity Criteria, version 4.0 was used to evaluate patients for toxicity (http://ctep.cancer.gov/protocolDevelopment/electronic_applications/ctc.htm).

### Statistical analysis

Descriptive statistics were used to characterize patients at study entry. Patient age and the number of cycles of treatment undergone were compared using the Mann-Whitney U test. Other patient demographics, response rates and toxicity rates were compared using Fisher’s exact test. The Kaplan-Meier method was used to characterize the PFS in terms of PSA response. P<0.05 was considered to indicate a statistically significant difference (two-sided).

## Results

### Patient characteristics

Patient characteristics are summarized in Table I. Baseline clinicopathological characteristics were generally well-balanced between the two groups and no significant difference between the two groups was observed.

### Efficacy

The treatment and efficacy are listed in Table II. One of the 20 patients in group B discontinued treatment by choice and was admitted to a hospice during the second week of the study. The most frequent reason for stopping treatment was disease progression. The PSA response rates were 65% in group A and 47.4% in group B among evaluated patients, and there was no statistically significant difference between the two groups (P=0.341).

**Figure 1:**
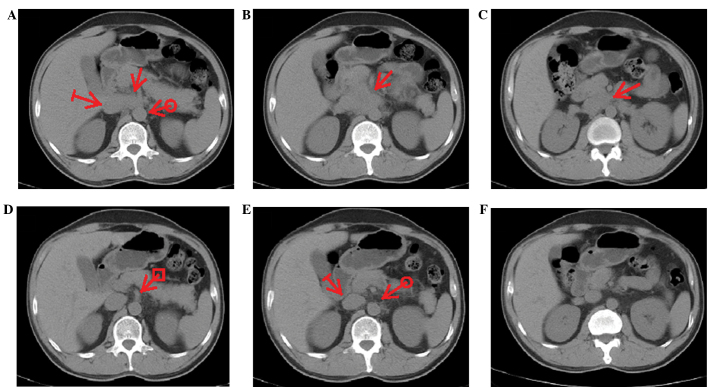
Following administration of docetaxel plus interferon-α for 6 months, a metastatic retroperitoneal lymph node tumor disappeared in the computed tomography scans of a 58-year-old male. (A–C) Pre-treatment image of retroperitoneal lymph node tumor (6 cm in diameter) located between the aorta and vena cava. The boundaries of the tumor and the vena cava are unclear (arrow, metastatic retroperitoneal lymph node; flat-shaped arrow, vena cava; circular arrow, aorta). (D–F) Post-treatment image demonstrates the disappearance of metastatic mass. The vena cava and aorta boundaries are clearly defined (square arrow, superior mesenteric artery).

In group A, among all the 20 patients with measurable lesions, 55% (11/20) achieved a tumor response according to the RECIST criteria. The patients with tumor response included seven for lymph node lesions, two for liver lesions and two for lung lesions. Notably, one patient displayed a complete response in group A (Fig. 1). This patient was a 58-year-old male with biopsy-proven retroperitoneal lymph node involvement (Fig. 1A–C). Six months after DTX plus IFN-α2b treatment, the PSA level had decreased from 755 ng/ml to an undetectable level and the retroperitoneal lymph node mass had disappeared (Fig. 1D–F). In group B, among all the 19 cases, 21.1% (4/19) of patients achieved a partial response. These responses were observed lymphnode and liver lesions (two patients) and lung lesions (two patients). However, no patients achieved a complete response in group B. The objective tumor response rates were 55% in group A and 21.1% in group B among evaluated patients, demonstrating a statistically significant difference between the two groups (P=0.048).

**Figure 2:**
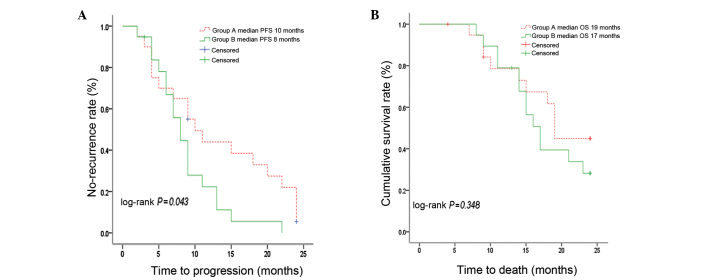
Kaplan-Meier estimate of (A) progression-free survival and (B) overall survival. PFS, progression-free survival; OS, overall survival; Group A, 75 mg/m^2^ DXT plus 3 mIU/m^2^ IFN-α2b; group B, 75 mg/m^2^ DXT alone; censored, (A) PSA progression did not occur in follow-up period; censored (B), patients were alive in the follow-up period.

Fig. 2A shows PFS by treatment group. The median PFS was 10 months (range 2–24; 95% CI, 5.98–14.02) for group A and 8 months (range 2–22; 95% CI, 5.94–10.06) for group B, with a 44±11 and 22±10% PFS rate at 1 year in the two groups, respectively. There was a statistically significant difference in PFS in groups A and B (P=0.043).

Fig. 2B shows the OS by treatment group. Median OS was 19 months (range 7–19; 95% CI, 17.64–20.37) for group A and 17 months (range 8–23; 95% CI, 14.33–19.67) for group B, with an overall survival rate of 45±12 and 28±11% at 2 years, respectively. There was no statistically significant difference in OS by treatment in the two groups (P=0.348).

### Toxicity

Table III shows the hematological and non-hematological toxicities stratified by grade and treatment group. In groups A and B, transient grade 3 to 4 neutropenia occurred in nine and six patients, grade 3 to 4 anemia occurred in three and five patients, and grade 3 to 4 general fatigue was observed in four and one patient(s), respectively. The proportion of patients with grade 3 to 4 toxicity displayed no statistically significant difference between the two groups (P>0.05).

## Discussion

The present study demonstrated that the tumor response rates in group A were significantly greater than those in group B. This difference may be due to the activity of IFN-α2b against CRPC. However, the rates of PAS and tumor response were 65 and 55%, respectively, which were higher than those observed in the study by DiPaola *et al* (23 and 15%, respectively) (20). We hypothesize that this difference between the trial by DiPaola *et al* and the present study was due to the following reasons: firstly, the DXT regimen was different, as DiPaola *et al* used DXT combined with IFN-α2b and 13-cis retinoic acid. We administered DXT combined with prednisone and IFN-α2b. DXT with prednisone has better efficacy and less toxicity, as has been confirmed by two large phase III clinical trials (PSA and tumor response rates were 44.4 and 44.2%, respectively) (3,4). Another reason is the difference in patient characteristics, particularly the metastatic sites. The present study had more patients with lymph node lesions than the study by DiPaola *et al*. A higher response of the lymph node lesions was also found in the study by van Haelst-Pisani *et al*(17); however, in this study, IFN-α2b was not combined with DXT and IFN-α2b toxicity is intolerable for patients administered high-dose IFN-α2b alone (10 mIU). In particular, one patient with a metastatic retroperitoneal lymph node (6 cm) achieved a complete tumor response in group A. In this patient, an undetectable PSA level and complete regression of disease persisted for more than 18 months. Complete disease regression was also observed in the study by Emerson and Morales (14), which used a low dosage of IFN-α2b. The PFS in group A was longer than that in group B. This difference may have resulted from the benefit of tumor response, as PFS included two responses, PSA and tumor, in our present study. These results demonstrated that IFN-α2b may expand the antitumor activity of DXT chemotherapy. Such increased activity may result from several mechanisms, which may not be mutually exclusive. Firstly, IFN-α2b may be able to reduce the DXT resistance by downregulating Bcl-2 as reported previously (11,21). Overexpression of Bcl-2 is important in antitumor drug resistance, including hormonal and chemotherapy resistance found in CRPC (22,23). Secondly, IFN-α2b may increase DXT sensitivity by upregulating the activity of p53 (12,24). Mutant p53 or the lack of functional p53 usually causes drug resistance, while restoring p53 function leads to regression of autochthonous lymphomas and sarcomas in mice without affecting normal tissues (9). In addition, IFN-α2b may synergize with DXT to promote apoptosis of tumor cells, the rapid growth of which depends on sustained angiogenesis (13).

Although the overall survival was longer in CRPC patients treated with DXT + IFN-α2b, there was no statistically significant difference between the two groups. One reason for this finding may be due to the fact that the improved PSA and tumor responses are not necessarily transformed into a survival advantage. For example, a large-scale phase III clinical study was recently conducted on the basis of the rationale that targeting neovasculature and microtubules could enhance the clinical impact of DXT alone (25). However, despite the advantages of improved PSA response and delayed disease progression, the anti-angiogenic agent bevacizumab in combination with DXT did not significantly prolong survival compared with DXT alone (25). The small sample size and short follow-up time of the present study may be another reason for these findings.

From the viewpoint of safety, while the incidence of grade 3 to 4 toxicity was not statistically significant between the two groups, neutropenia was still frequently observed in group A. This side-effect may be due to suppression of the release of granulocytes by IFN-α2b in the bone marrow (26). This adverse event can often be safely and effectively controlled by administration of G-CSF. It should be noted that five patients with grade 3 to 4 neutropenia were poorly responsive to G-CSF; however, neutropenia could be attenuated by reducing the DXT dose, increasing the interval between doses and decreasing the number of chemotherapy cycles. Thus, careful follow-up is required. However, a few reports have shown that the toxicity of IFN-α2b is not well-tolerated in CRPC (20,27). We hypothesize that the toxicities in the previous studies and the present study are different due to the following reasons. First, the IFN-α2b dosage was different. We employed a lower dose of IFN-α2b [3 vs. 6 mIU/m^2^(20) or 10 mIU/m^2^(17)]. The second reason is the differences in infusion time of IFN-α2b. DXT-induced hematological toxicities most frequently occurred during the first 10 days of every cycle, particularly on days 5 and 7 (28). DXT combined with IFN-α2b may increase the patient’s adverse events during this period. Patients were administered in the third week of the cycle (days 16, 18 and 20) in the present study. However, IFN-α2b was used on days 1 and 2 of each week in the trial by DiPaola *et al*(20). In addition, the patients had a better performance status prior to chemotherapy [ECOG ≤1 vs. ECOG ≤2 (17,19)] in the present study.

In conclusion, a low dosage of IFN-α2b may expand the effects of DXT chemotherapy with improvements in tumor response and PFS in patients with CRPC. The main side-effects were neutropenia, fever and general fatigue, which may be safely controlled by administration of G-CSF and symptomatic treatment. Further evaluation of a large number of patients with a longer follow-up period is required.

## Figures and Tables

**Table I:** Patient characteristics.

Index	Group A, n=20	Group B, n=20	P-value
Age (years)			
Median (range)	68 (58–77)	66 (62–76)	0.642
ECOG performance score, n (%)			
0	11 (55)	7	0.341
1	9 (45)	13	0.200
Prior treatment, n (%)			
Prostatectomy + hormonal therapy	5 (25)	7 (35)	0.501
Radiotherapy + hormonal therapy	13 (65)	11 (55)	0.748
Hormonal therapy	2 (10)	2 (10)	1.000
Site of metastasis, n (%)			
Bone	18 (90)	19 (95)	0.501
Lymph node	10 (50)	8 (40)	0.748
Liver	3 (10)	2 (10)	0.695
Lung	6 (30)	8 (40)	1.000
PSA			
Median (range)	49.05 (10.84–1328.53)	63.27 (12.20–1324.48)	0.317
Biopsy Gleason score, n (%)			
≤6	8 (40)	10 (50)	0.751
7	6 (30)	3 (15)	0.451
8–10	6 (30)	7 (35)	1.000
Time from start of ADT to CRPC (months)			
Median (range)	17.50 (7.45–67.16)	15.00 (6.68–50.43)	0.131

Group A, 75 mg/m^2^ DXT plus 3 mIU/m^2^ IFN-α2b; group B, 75 mg/m^2^ DXT alone; ECOG, Eastern Cooperative Oncology Group; PSA, prostat-specific antigen; ADT, androgen deprivation therapy; CRPC, castration-resistant prostate cancer.

**Table II:** Treatment and efficacy.

Index	Group A	Group B	P-value
No. of cycles (months)			
Median (range)	8 (3–12)	9 (2–12)	0.573
Dose reduction (%)	15 (3/20)	10.5 (2/19)	1.000
PSA response (%)	65 (13/20)	47.4 (9/19)	0.341
Objective tumor response	55 (11/20)	21.1 (4/19)	0.048
PFS (months)			
Median (range)	10 (2–24)	8 (2–22)	0.043
Overall survival (months)			
Median (range)	19 (7–19)	17 (8–23)	0.348

Group A, 75 mg/m^2^ DXT plus 3 mIU/m^2^ IFN-α2b; group B, 75 mg/m^2^ DXT alone; PSA, prostate-specific antigen; PFS, progression-free survival.

**Table III:** Treatment-related toxicity.

	Group A, n=20		Group B, n=19		
			
Toxicity	Grade 1–2	Grade 3–4	Grade 1–2	Grade 3–4	P-value (grade 3–4)
Neutropenia, n (%)	7 (35)	9 (45)	8 (40)	6 (30)	0.514
Anemia	7 (35)	3 (15)	8 (40)	5 (25)	0.451
Febrile neutropenia	3 (15)	0	5 (25)	0	
Platelets	2 (10)	0	0	0	
Fever	3 (15)	0	3 (15)	0	
Chills	2 (10)	0	4 (20)	0	
Allergic reaction	0	0	1 (5)	0	
Nausea/vomiting	2 (10)	0	3 (15)	0	
Diarrhea	4 (20)	0	2 (10)	0	
General fatigue	1 (5)	4 (20)	2 (10)	1 (5)	0.342
Edema	2 (10)	0	3 (15)	0	
Liver dysfunction	2 (10)	0	4 (20)	0	

Group A, 75 mg/m^2^ DXT plus 3 mIU/m^2^ IFN-α2b; group B, 75 mg/m^2^ DXT alone.
